# Caspase cleavage of GFAP produces an assembly-compromised proteolytic fragment that promotes filament aggregation

**DOI:** 10.1042/AN20130032

**Published:** 2013-11-19

**Authors:** Mei-Hsuan Chen, Tracy L. Hagemann, Roy A. Quinlan, Albee Messing, Ming-Der Perng

**Affiliations:** *Institute of Molecular Medicine, College of Life Sciences, National Tsing Hua University, Hsinchu, Taiwan.; †Waisman Center, University of Wisconsin, WI, USA; ‡School of Biological and Biomedical Sciences, The University of Durham, UK; §Department of Comparative Biosciences, University of Wisconsin, WI, USA

**Keywords:** Alexander disease, caspase, GFAP, intermediate filament, AD, Alzheimer’s disease, AxD, Alexander disease, C-GFAP, C-terminal GFAP, CNS, central nervous system, DTT, dithiothreitol, GAPDH, glyceraldehyde-3-phosphate dehydrogenase, GFAP, glial fibrillary acidic protein, IF, intermediate filament, N-GFAP, N-terminal GFAP OA, okadaic acid, TBS, Tris-buffered saline, TBST, TBS containing 0.1% (v/v) Tween 20

## Abstract

IF (intermediate filament) proteins can be cleaved by caspases to generate proapoptotic fragments as shown for desmin. These fragments can also cause filament aggregation. The hypothesis is that disease-causing mutations in IF proteins and their subsequent characteristic histopathological aggregates could involve caspases. GFAP (glial fibrillary acidic protein), a closely related IF protein expressed mainly in astrocytes, is also a putative caspase substrate. Mutations in GFAP cause AxD (Alexander disease). The overexpression of wild-type or mutant GFAP promotes cytoplasmic aggregate formation, with caspase activation and GFAP proteolysis. In this study, we report that GFAP is cleaved specifically by caspase 6 at VELD^225^ in its L12 linker domain *in vitro*. Caspase cleavage of GFAP at Asp^225^ produces two major cleavage products. While the C-GFAP (C-terminal GFAP) is unable to assemble into filaments, the N-GFAP (N-terminal GFAP) forms filamentous structures that are variable in width and prone to aggregation. The effect of N-GFAP is dominant, thus affecting normal filament assembly in a way that promotes filament aggregation. Transient transfection of N-GFAP into a human astrocytoma cell line induces the formation of cytoplasmic aggregates, which also disrupt the endogenous GFAP networks. In addition, we generated a neo-epitope antibody that recognizes caspase-cleaved but not the intact GFAP. Using this antibody, we demonstrate the presence of the caspase-generated GFAP fragment in transfected cells expressing a disease-causing mutant GFAP and in two mouse models of AxD. These findings suggest that caspase-mediated GFAP proteolysis may be a common event in the context of both the GFAP mutation and excess.

## INTRODUCTION

IFs (intermediate filaments), together with microtubules and microfilaments, form an interconnected network of the cytoskeleton, which gives cells their form, shape and functions. In humans, at least 70 different IF proteins have been identified (Hesse et al., 2001), and there is a complex expression pattern of IF proteins specific for every cell type. This is well demonstrated in astrocytes, in which GFAP (glial fibrillary acidic protein), vimentin and nestin are the major IF proteins. While vimentin and nestin are mainly expressed in immature astrocytes, GFAP is coexpressed with vimentin in mature astrocytes (Eliasson et al., 1999).

AxD (Alexander disease) is a primary genetic disorder of astrocytes that typically affects young children (Messing et al., 2012). It is caused by missense mutations in the coding region of *GFAP* (Brenner et al., 2001). Although this is the most common form of AxD, milder forms with intermediate ages of onset also exist. A key histopathological feature of all forms of AxD is the widespread deposition of inclusion bodies within astrocytes known as Rosenthal fibers, consisting of aggregated GFAP, the small stress proteins HSP27 and αB-crystallin (Tomokane et al., 1991; Head et al., 1993; Iwaki et al., 1993) and likely other unidentified proteins. Whether Rosenthal fibres *per se* cause astrocyte dysfunction and what the precise trigger is for their formation are not clear. Mouse models created via both transgenic and knock-in approaches (Messing et al., 1998; Hagemann et al., 2006; Tanaka et al., 2007) clearly show that simply elevating the level of wild-type GFAP or expressing mutant GFAP leads to the formation of Rosenthal fibres. Astrocytes cultured from these mice exhibit decreased cell proliferation and increased caspase activity (Cho and Messing, 2009). Similar observations were made in transfected cell lines, where the expression of mutant forms of GFAP causes extensive filament aggregation, with caspase activation and GFAP cleavage (Chen et al., 2011). These findings are of interest because they represent some of the first indications of a direct link between abnormal protein aggregation and GFAP proteolysis through caspase activation.

Caspases are a family of cysteine proteases that specifically cleave target proteins at sites next to aspartic acid residues (Pop and Salvesen, 2009). Caspase cleavage of several IF proteins, including nuclear lamins (Orth et al., 1996; Rao et al., 1996; Takahashi et al., 1996; Ruchaud et al., 2002), keratins (Caulin et al., 1997; Ku et al., 1997; Ku and Omary, 2001; Tao et al., 2008), desmin (Chen et al., 2003) and vimentin (Morishima, 1999; Byun et al., 2001; Nakanishi et al., 2001), leads to the destruction of the nuclear envelope and the disassembly of the cytoplasmic IF network that characterize apoptosis. Each of these IF proteins is cleaved by caspase 6 at a consensus site in the L12 linker region of the rod domain, although cleavage by other caspases at additional sites also occurs (Marceau et al., 2007). Caspase 6 is an executioner caspase based on its role in cleavage of nuclear structural proteins (Orth et al., 1996; Hirata et al., 1998) and its requirement for activation by upstream initiator caspases (Boatright and Salvesen, 2003). Apart from its executive role, caspase 6 can also cleave and activate other caspases (Slee et al., 1999; Cowling and Downward, 2002), such as caspase 3 (Allsopp et al., 2000; Graham et al., 2010). Although the precise trigger for the activation of caspase 6 is not clear, emerging data suggest a role for its activation in neurodegenerative conditions (Graham et al., 2011). Caspase 3 activation and GFAP cleavage contribute to the damaged astrocytes in AD (Alzheimer's disease) brain (Mouser et al., 2006). In addition, a proteomic approach identified GFAP as a potential substrate of caspase 6 in human primary neurons (Klaiman et al., 2008). Although GFAP is itself a caspase substrate, caspase-mediated cleavage of GFAP in astrocytes has not been fully explored, and the assembly properties of the caspase cleavage products have not previously been addressed.

Here, we report that GFAP is specifically cleaved by caspase 6 *in vitro*. Mutagenesis analysis coupled with an *in vitro* caspase cleavage assay confirmed that VELD^225^ in the L12 linker domain of GFAP is the major caspase cleavage site. Caspase cleavage of GFAP produces an N-terminal cleavage product (N-GFAP) that significantly perturbs *in vitro* filament assembly and affects normal filament assembly in a way that promotes inter-filament interactions. In addition, transient transfection studies demonstrate that the overexpression of N-GFAP induces the formation of GFAP aggregates that also disrupt the endogenous networks of intact GFAP in transfected human astrocytoma cells. Furthermore, a neo-epitope antiserum specific to N-GFAP reveals the presence of the caspase-cleaved GFAP fragment in cells expressing disease-causing mutant GFAP and in two types of AxD models that have previously been shown to have varying levels of GFAP accumulation in different regions of the CNS (central nervous system) (Messing et al., 1998; Hagemann et al., 2006). These results imply that caspase-mediated cleavage of GFAP correlates with elevated GFAP in the context of GFAP mutation and accumulation. Moreover, we provide evidence to suggest that caspase cleavage of GFAP has important functional consequences, decreasing GFAP filament solubility by changing filament–filament interactions in a way that promotes aggregation.

## MATERIALS AND METHODS

### Plasmid construction and site-directed mutagenesis

Mutant GFAP constructs with specifically altered amino acids were generated by site-directed mutagenesis with full-length human *GFAP* (GenBank accession no. J04569) in the pcDNA3 vector (Invitrogen) as a template (Perng et al., 2006). All newly generated DNA constructs were verified by sequencing before use. To generate the amino-terminal caspase-cleavage product (amino acids 1–225, designated N-GFAP), a translational stop codon right after the glutamic acid of the VELD^225^ sequence was introduced by site-directed mutagenesis with 5′-GTGGAGCTTGACTAGGCCAAGCCAG-3′ and 5′- CTGGCTTGGCCTAGTCAAGCTCCAC-3′ as forward and reverse primers, respectively. The C-terminal fragment of GFAP (amino acids 226–432, designated C-GFAP (C-terminal GFAP)) was PCR-amplified from the human *GFAP* cDNA using the following oligonucleotides: 5′-CATATGGCCAAGCCAGACCTCACCGC-3′ and 5′-GAATTCTCACATCACATCCTTGTGC-3′. The amplified PCR product containing C-GFAP was cloned into the pJET cloning vector (Thermo Scientific), and the sequence was confirmed by DNA sequencing. The Myc epitope was added to the N-terminal end of human *GFAP* by site-directed mutagenesis using the oligonucleotide GAACAAAAACTCATCTCAGAAGAGGATCTG. For expression in bacteria, cDNAs of full-length GFAP and its variants were subcloned into the pET23b vector (EMD Millipore) with use of the *Nde*I and *Eco*RI restriction sites.

### Expression and purification of recombinant GFAPs

For bacterial expression of proteins, pET23b vector containing cDNAs of either intact GFAP or its variants were transformed into the *Escherichia coli* BL21(DE3) pLysS strain (EMD Millipore). Overexpressed GFAP formed inclusion bodies, which were prepared as described previously (Perng et al., 2006). The expressed proteins were further purified by ion-exchange chromatography using an AKTAprime plus system (GE Healthcare) equipped with a DEAE Sepharose column (GE Healthcare). Column fractions were analyzed by SDS/PAGE followed by Coomassie Blue staining, and those containing purified proteins were collected and stored at −80°C. Protein concentrations were determined by BCA Protein Assay Kit (Thermo Scientific) with use of BSA as standard. Recombinant mouse GFAP (Ralton et al., 1994) and human vimentin (Perng et al., 2004) were purified as described previously.

### Caspase cleavage of GFAP *in vitro*

Purified human recombinant GFAP (1 μg) was diluted in caspase assay buffer (50 mM HEPES, pH 7.2, 50 mM NaCl, 0.1% (w/v) CHAPS, 10 mM EDTA, 10 mM DTT (dithiothreitol) and 5% (v/v) glycerol) in the absence or presence of active recombinant human caspase 3 or caspase 6 (BioVision) at a final concentration of 0.25 U/μl. After incubation for 1 h at 37°C, the cleavage products were separated by SDS/PAGE, followed by Coomassie Blue staining or immunoblotting. To determine the caspase cleavage site in GFAP, caspase-cleaved GFAP was separated by SDS/PAGE, transferred to a PVDF membrane (EMD Millipore), stained with Coomassie Blue and then subjected directly to N-terminal sequencing (Mission Biotech). To determine whether the D225E mutant GFAP was resistant to caspase cleavage *in vitro*, purified mutant GFAP was incubated with recombinant active caspase 6 and analyzed as above. The proteolytic activities of caspase 3 and caspase 6 were confirmed using a structurally related IF protein, vimentin (Byun et al., 2001), as a positive control.

### *In vitro* assembly and sedimentation assay

Purified GFAP was diluted to 0.3 mg/ml in 6 M urea in a buffer of 10 mM Tris/HCl, pH 8, 5 mM EDTA, 1 mM EGTA and 1 mM DTT. Protein samples were dialyzed stepwise against the same buffer containing 3 M urea for 6 h and then against the same buffer without urea at 4°C overnight. Filament assembly was completed by dialyzing against assembly buffer (10 mM Tris/HCl, pH 7.0 and 50 mM NaCl) for 12–16 h at room temperature. The filament-forming efficiency was assessed by high-speed sedimentation assay as described previously (Nicholl and Quinlan, 1994). In brief, the assembly mixture was layered onto a cushion of 0.85 M sucrose in the final assembly buffer and centrifuged for 30 min at 80000 ***g*** at 20°C in a Hitachi S55S rotor using a CS150NX tabletop micro-ultracentrifuge (Hitachi Koki Co.). To investigate the extent of filament–filament interactions in the whole filament population, samples were subjected to low-speed centrifugation at 3000 ***g*** for 5 min at room temperature in a benchtop centrifuge (Eppendorf). The supernatant and pellet dissolved in Laemmli's sample buffer (Laemmli, 1970) in volumes proportional to the original sample size were analyzed by SDS/PAGE and were visualized by Coomassie Blue staining. In some instances, the proportions of GFAP distributed between pellet and supernatant fractions were measured using an image analyzer (ImageQuant 350, GE Healthcare). Coomassie Blue signals for individual bands were quantified using the image analysis software (ImageQuant TL 7.0, GE Healthcare).

### Electron microscopy

GFAP was diluted in assembly buffer to 0.1 mg/ml and was negatively stained with 1% (w/v) uranyl acetate (Electron Microscopy Sciences). Samples were spread on carbon-coated copper grids (Ted Pella Inc.) and examined with an HT7700 transmission electron microscope (Hitachi High-Technologies) with an accelerating voltage of 100 kV. Images were acquired using a CCD (charge-coupled-device) camera before being further processed in Adobe Photoshop CS II (Adobe Systems). Filament length and diameter were measured on enlarged electron micrographs using ImageJ software (National Institutes of Health).

### Cell cultures, transfection and treatments

Human breast cancer epithelial MCF7 cells were obtained from the European Collections of Cell Cultures (Sigma-Aldrich). Human astrocytoma U343MG cells were provided by Dr. J. T. Rutka (Division of Neurosurgery, University of Toronto, Toronto, Canada) and were grown essentially as described (Perng et al., 2006). For transient transfection studies, plasmid DNA was prepared using a PureLink™ HiPure Midi-Prep kit (Invitrogen). Cells on 13 mm coverslips at a density of 40–50% confluency were transfected using GeneJuice® transfection reagent (EMD Millipore) according to the manufacturer's instructions. Cells were allowed to recover for 48 h before further processing for immunofluorescence microscopy. In some experiments, transfected cells were treated with apoptosis-inducing drug OA (okadaic acid; Enzo Life Sciences) at a final concentration of 200 nM for 2–4 h or the general caspase inhibitor zVAD-fmk (Promega) at a final concentration of 40 μM.

### Generation of caspase cleavage site-directed antibody

An immunogen peptide, ARQQVHVELD^225^, corresponding to amino acids 216–225 of human GFAP, was synthesized, coupled to keyhole limpet hemocyanin and used for immunization (Yao-Hong Biotechnology Inc.). The rabbit serum providing the highest titer and specificity was subsequently used. The caspase cleavage site-specific antibody was further purified from rabbit serum by affinity chromatography. Briefly, CnBr-activated Sepharose 4B beads (GE Healthcare) were resuspended in 1 mM HCl for 30 min. After being washed twice with coupling buffer (0.1 M sodium carbonate, pH 8.3), swelled beads were mixed with 10 mg immunogen peptide and rotated at 4°C for approximately 16 h. The beads were washed with coupling buffer and blocked in 1 M glycine, pH 8 for 2 h at 4°C. Rabbit antiserum was then loaded onto the peptide column, from which the antigen-specific antibody was eluted with 0.1 M glycine (pH 2.5) into tubes containing 1 M Tris/HCl (pH 9.3). Fractions were analyzed by SDS/PAGE, and those containing antibodies were collected and tested for their specificity by immunoblotting and immunofluorescence microscopy.

### Immunofluorescence microscopy

Cells grown to about 80% confluency on glass coverslips were processed for indirect immunofluorescence microscopy essentially as described (Perng et al., 2006). The primary antibodies used in this study were mouse monoclonal anti-GFAP antibodies GA-5 (1:500, Sigma-Aldrich) and SMI-21 (1:500, Covance) and rabbit polyclonal anti-GFAP (DakoCytomation) and anti-active caspase 6 (BioVision) antibodies. Primary antibodies were detected using Alexa Fluor® 488 (1:600)- or Alexa Fluor® 594 (1:600)-conjugated secondary antibodies (Invitrogen). The glass coverslips were mounted on slides and observed using a Zeiss LSM510 laser scanning microscope using a ×40 Plan-Neofluar objective (NA 0.75) (Carl Zeiss). Images were collected in multi-track mode by LSM510 software taking 1.0 μm optical sections and processed for figures using Adobe® Photoshop CS II (Adobe System).

### Cell fractionation, immunoblotting and immunoprecipitation

Cells grown on 10-cm^2^ Petri dishes were transfected with control vector (pcDNA3.1) or the same vector encoding the indicated GFAP variants. At 48 h after transfection, cells were lysed using harsh extraction buffer [10 mM Tris/HCl pH 7.6, 140 mM NaCl, 5 mM EDTA, 1 mM EGTA, 1% (v/v) Triton X-100, 0.5% (w/v) sodium deoxycholate, 0.1% (w/v) SDS] supplemented with 1% (v/v) protease inhibitor cocktail (Sigma-Aldrich) and 1 mM PMSF containing 250 U/ml benzonase nuclease (EMD Millipore) as described previously (Perng et al., 2006). Cell lysates were then homogenized in a 1 ml Dounce homogenizer (Wheaton). After protein concentration determination, total cell lysates were equalized by adding appropriate volumes of Laemmli's sample buffer (Laemmli, 1970) before analyzing by SDS/PAGE and immunoblotting. To prepare supernatant and pellet fractions, total cell lysates were centrifuged at 16000 ***g*** for 10 min at 4°C in a benchtop centrifuge (Eppendorf). The resulting pellets were resuspended in Laemmli's sample buffer in a volume that was equivalent to the supernatant.

Immunoblotting was performed using the semidry blotting method according to the manufacturer's specifications (Bio-Rad) and modified as described (Perng et al., 2006). Primary antibodies used were monoclonal anti-GFAP GA-5 (1:5000, Sigma-Aldrich) and SMI-21 (1:5000, Covance), anti-Myc (9E10, 1:5000, Sigma-Aldrich), anti-vimentin (1:5000, V9, Sigma-Aldrich), anti-caspase 3 (1:1000, Cell Signaling Technology) and anti-actin (1:5000, AC-40, Sigma-Aldrich) antibodies. Primary antibodies were detected by HRP (horseradish peroxidase)-conjugated secondary antibodies (DakoCytomation) diluted by 1:5000 in blocking buffer containing 3% (w/v) BSA in TBS (Tris-buffered saline), followed by washing with TBS for a total of 30 min with several changes. Antibody labeling was detected by ECL (enhanced chemiluminescence; Western Lightning Plus-ECL, PerkinElmer) using a luminescent image analyzer (ImageQuant 350, GE Healthcare). The strength of signals was quantified using the image analysis software (ImageQuant TL 7.0, GE Healthcare).

For immunoprecipitation, insoluble proteins in the pellet fraction were first solubilized in 10 mM Tris/HCl, pH 8 and 1 mM EDTA containing 1% (w/v) SDS and then diluted 1:10 in harsh extraction buffer without SDS. The resulting solution was precleared by incubating with a 50% (v/v) slurry of protein A Sepharose (GE Healthcare) for 1 h at 4°C. The precleared sample was then incubated with the GFAP fragment-specific D225 antibody for 1 h at 4°C, followed by the capture of immunocomplexes by protein A-Sepharose beads at 4°C overnight. Immunoprecipitates were washed three times with harsh extraction buffer, resuspended in Laemmli's sample buffer and analyzed by immunoblotting.

### Animal use and ethics statement

Knock-in mice carrying the R236H mutation in mouse *Gfap* (Hagemann et al., 2006) and transgenic mice (GFAP^Tg^) expressing added copies of a human *GFAP* transgene (Messing et al., 1998) were generated as described previously. GFAP knock-in mice (GFAP**^+^**^/R236H^) were maintained as heterozygotes in the 129S6 background and GFAP transgenic mice (GFAP^Tg^) were maintained as hemizygotes in the FVB/N background. All experiments involving animals were approved by the Institutional Animal Care and Use Committee of the Graduate School at the University of Wisconsin-Madison.

### Analyses of GFAP expression and proteolysis in mouse models of AxD

For total protein preparation, brain samples were homogenized in SDS lysis buffer (50 mM, Tris/HCl (pH 7.4), 5 mM EDTA, 2% (w/v) SDS, 1 mM Pefabloc SC (Sigma-Aldrich) and Complete™ Mini protease inhibitor mixture (Roche Applied Science) using a Geno/Grinder tissue homogenizer (SPEX CertiPrep). After homogenization, samples were boiled for 15 min and then diluted in PBS. Protein concentration was determined using the BCA Protein Assay kit (Thermo Scientific) with BSA as a standard. To prepare soluble and insoluble fractions, brain tissues were first homogenized in deoxycholate lysis buffer (20 mM, Tris/HCl (pH 7.5), 150 mM NaCl, 1 mM EDTA, 1% (w/v) Triton X-100, 0.5% (w/v) sodium deoxycholate, 1 mM Pefabloc SC and Complete™ Mini protease inhibitor mixture), followed by centrifugation at 16000 ***g*** for 20 min at 4°C. The supernatant was collected as the soluble fraction, and the remaining pellet, representing the insoluble fraction, was resuspended in urea buffer (7 M urea, 50 mM Tris/HCl, pH 7.5, 10 mM NaCl, 1 mM EDTA, 1 mM Pefabloc SC and Complete™ Mini protease inhibitor mixture). After the protein concentration determination, protein samples were electrophoresed on 10% (w/v) Criterion™ Precast Gels (Bio-Rad), followed by transfer to Immobilon-FL membranes (EMD Millipore). The membranes were incubated with mouse monoclonal anti-GFAP GA-5 (1:10,000, Sigma-Aldrich) and rabbit polyclonal caspase cleavage site-specific D225 antibodies (1:5,000) for 1 h at room temperature. After being washed with TBST (TBS containing 0.1% (v/v) Tween 20), membranes were incubated for 2 h with IRDye 680- or 800-conjugated secondary antibody (LI-COR Biosciences) diluted by 1:10,000 in TBST. Immunoblots were analyzed with an Odyssey Infrared Imaging System (LI-COR Biosciences), and the signal intensity of proteins of interest was quantified using the Image Studio software (Ver. 2.0, LI-COR Biosciences). Equal loading of each fraction was verified using a polyclonal anti-GAPDH (glyceraldehyde-3-phosphate dehydrogenase) antibody (Abcam).

### ELISA for GFAP

For quantification of GFAP, a sandwich ELISA was carried out as described previously (Hagemann et al., 2009; Hagemann et al., 2012) using the monoclonal anti-GFAP antibody cocktail SMI-26 (Covance) as capture antibodies and a rabbit polyclonal anti-GFAP antibody (DakoCytomation) as a detecting antibody.

## RESULTS

### Specific cleavage of GFAP at Asp^225^ by caspase 6 *in vitro*

**Figure 1:**
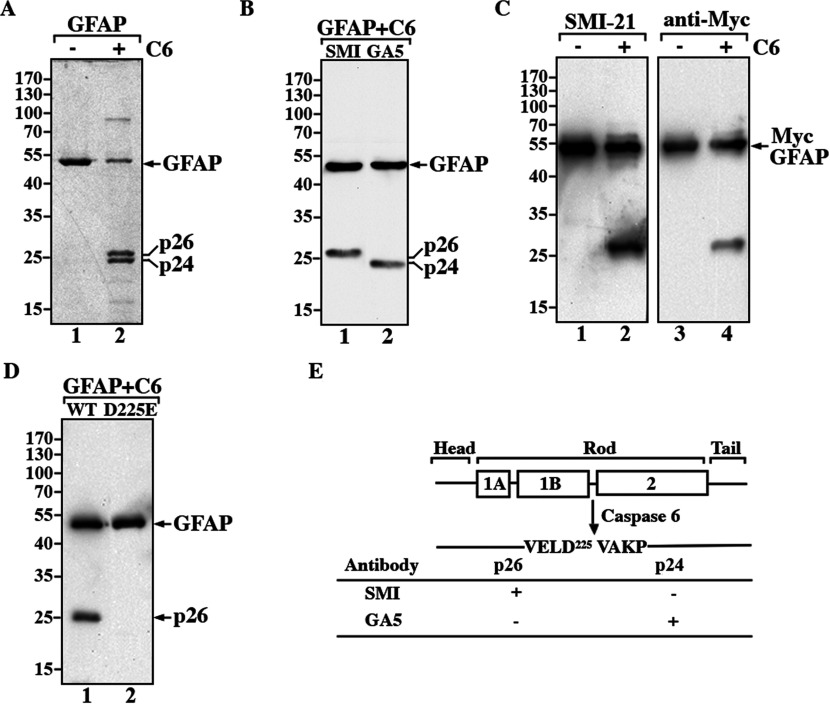
GFAP is specifically cleaved at Asp^225^ by caspase 6 *in vitro* (**A**) Purified recombinant human GFAP was either untreated (lane 1) or treated with 2.5 U of active caspase 6 (lane 2) for 1 h at 37°C. The reaction products were separated by SDS/PAGE, followed by Coomassie Blue staining. (**B**) GFAP cleaved by active caspase 6 generated two prominent proteolytic fragments, p26 and p24 (A, lane 2), which were recognized by the anti-GFAP antibodies SMI-21 (lane 1) and GA-5 (lane 2), antibodies, respectively. Both antibodies also recognized intact GFAP (lanes 1 and 2). (**C**) Caspase cleavage of Myc-GFAP generated a proteolytic fragment that was recognized by both the anti-GFAP SMI-21 (lane 2) and anti-Myc (lane 4) antibodies. (**D**) The D225E mutant GFAP was resistant to caspase 6 cleavage (lane 2), whereas caspase cleavage of wild-type GFAP generated appropriately sized proteolytic products (lane 1). The molecular mass markers (in kDa) are indicated at the left of each panel. (**E**) A schematic view of the structural organization and caspase-mediated digestion of GFAP. GFAP comprises a central α-helical rod domain, flanked by non-helical head and tail domains (denoted by black bars). Within this rod domain, subhelical segments (denoted by boxes) are connected by short linker sequences (denoted by black bars). p26 and p24 represent the major caspase-cleaved GFAP fragments that migrate on SDS/PAGE with apparent molecular masses of 26 and 24 kDa, respectively. The single-letter amino acid codes for the caspase cleavage site are also indicated. The ability of the anti-GFAP antibodies to detect specific GFAP fragments is summarized. (+), immunopositive; (−), immunonegative.

Upon incubation of recombinant human GFAP with caspase 6, we found that GFAP can be cleaved to generate two major degradation products of about 26 and about 24 kDa, respectively (Figure 1A, lane 2, indicated as p26 and p24). Immunoblotting analysis with two commercially available monoclonal antibodies revealed that SMI21 antibody recognized the p26 fragment (Figure 1B, lane 1), whilst the GA-5 antibody recognized the p24 fragment (Figure 1B, lane 2). As the epitope of the GA-5 antibody resides in the tail domain of GFAP (Chen et al., 2011), this suggested that the p24 fragment may correspond to the C-GFAP. SMI-21 antibody detected the p26 fragment of N-terminally tagged GFAP (Myc-GFAP; Figure 1C). Attempts to sequence the amino terminus of N-GFAP by automated Edman degradation were unsuccessful, likely due to a blocked N-terminus as has been demonstrated on desmin (Mavroidis et al., 2008). Nevertheless, C-GFAP sequencing revealed ^226^VAKP in the amino terminus of this fragment, suggesting that VELD^225^ at the carboxy end of N-GFAP is the caspase 6 cleavage site. This was confirmed by substituting aspartate with glutamate (D225E) in the predicted recognition site. D225E GFAP was not cleaved by caspase 6 (Figure 1D). These data unambiguously demonstrate that GFAP is cleaved specifically by caspase 6 at Asp^225^ in its L12 linker domain *in vitro* (Figure 1E).

### Assembly properties of the caspase-generated cleavage products

**Figure 2:**
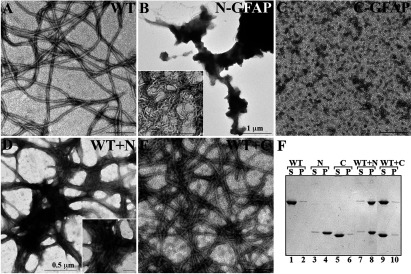
Assembly properties of the caspase-cleaved GFAP fragments Purified N-, C- and intact GFAP at a concentration of 0.3 mg/ml were assembled *in vitro*. The assembly products were negatively stained and visualized by electron microscopy. Intact GFAP assembled into typical 10-nm filaments (**A**), whereas N-GFAP formed irregular filamentous structures (**B**, inset) that had a strong tendency to aggregate (**B**). C-GFAP failed to assemble into extended filaments but instead formed discrete particles (**C**). When C-GFAP was coassembled with intact GFAP, the filaments formed were not dramatically altered in morphology (**E**) compared with those made from intact GFAP alone (**A**). In contrast, mixing N-GFAP in a 25%:75% ratio with intact GFAP resulted in the formation of filaments (**D**, inset) that had a strong tendency to aggregate (**D**). Bar, 200 nm, except in panels B and D, where the bars are 1 μm and 0.5 μm, respectively. The extent of filament aggregation was assessed by a low-speed sedimentation assay (**F**). Intact, N- and C-GFAP were assembled either alone or in combinations of intact GFAP with either N-GFAP or C-GFAP at a 75%:25% ratio. Assembly products were subjected to low-speed centrifugation, and the supernatant (S) and pellet (P) fractions were analyzed by SDS/PAGE, followed by Coomassie Blue staining. Most of the assembled intact GFAP remained in the supernatant fraction (lane 1), whereas N-GFAP was found mainly in the pellet fraction (lane 4). Coassembly of N-GFAP with full-length protein resulted in formation of assembly complexes that were easily sedimented into the pellet fraction (lane 8). In contrast, C-GFAP, either on its own (lane 5) or in combination with intact GFAP (F, lane 9), remained in the supernatant fraction.

We were then interested to know the assembly properties of N-GFAP and C-GFAP. Like other type III IF proteins, *in vitro* assembly of GFAP goes through several distinct and well-characterized stages (Herrmann and Aebi, 2004). The first stage is the formation of the ULFs (unit length filaments), which anneal end to end longitudinally and then compact radially to form the mature filament that is 10 nm wide and many microns long. Under standard assembly conditions, wild-type GFAP formed typical 10 nm filaments that were many microns in length (Figure 2A). N-GFAP formed large aggregates (Figure 2B), which at higher magnification could be seen to contain irregular filamentous structures (Figure 2B, inset). C-GFAP failed to self-assemble into filaments but instead formed discrete particles approximately 12–20 nm in diameter (Figure 2C).

The impact of both fragments upon the assembly of intact wild-type GFAP was also assessed. N-GFAP affected the assembly in a concentration dependent manner (Figure 2D), whereas C-GFAP did not apparently change the assembly or filament morphology of intact GFAP (Figure 2E). 10% N-GFAP with 90% intact GFAP did not dramatically alter the morphology of the assembled filaments (Supplementary Figure S1 available at http://www.asnneuro.org/an/005/an005e125add.htm) compared with those made from 100% intact GFAP (Figure 2A). Increasing the proportion of N-GFAP to 25% produced filaments that had a strong tendency to associate laterally (Figure 2D and inset). These data suggest that N-GFAP may integrate into GFAP filaments, but at the expense of promoting filament–filament interactions. Therefore, we performed a low-speed sedimentation assay to assess the extent of filament–filament interactions in the whole filament population. Intact GFAP remained largely (95%) in the supernatant fraction (Figure 2F, lane 1) and it had assembled efficiently (Supplementary Figure S2 available at http://www.asnneuro.org/an/005/an005e125add.htm). Including 25% N-GFAP in the assembly of intact GFAP (Figure 2F, lane 8, labeled P) produced a dramatic change in the sedimentation behavior of the intact GFAP. The N-GFAP mostly pelleted when assembled on its own (Figure 2F, lane 4), whilst C-GFAP remained mostly (90%) in the supernatant fraction (Figure 2F, lane 5). Indeed coassembly of C-GFAP with intact GFAP, even at a 50%:50% ratio (Figure 2F, lane 10) did not change the sedimentation characteristics of the assembled material. These data support the suggestion that the integration of N-GFAP into GFAP filaments resulted in increased filament–filament interactions.

### Generation and characterization of site-directed caspase cleavage antibody

**Figure 3:**
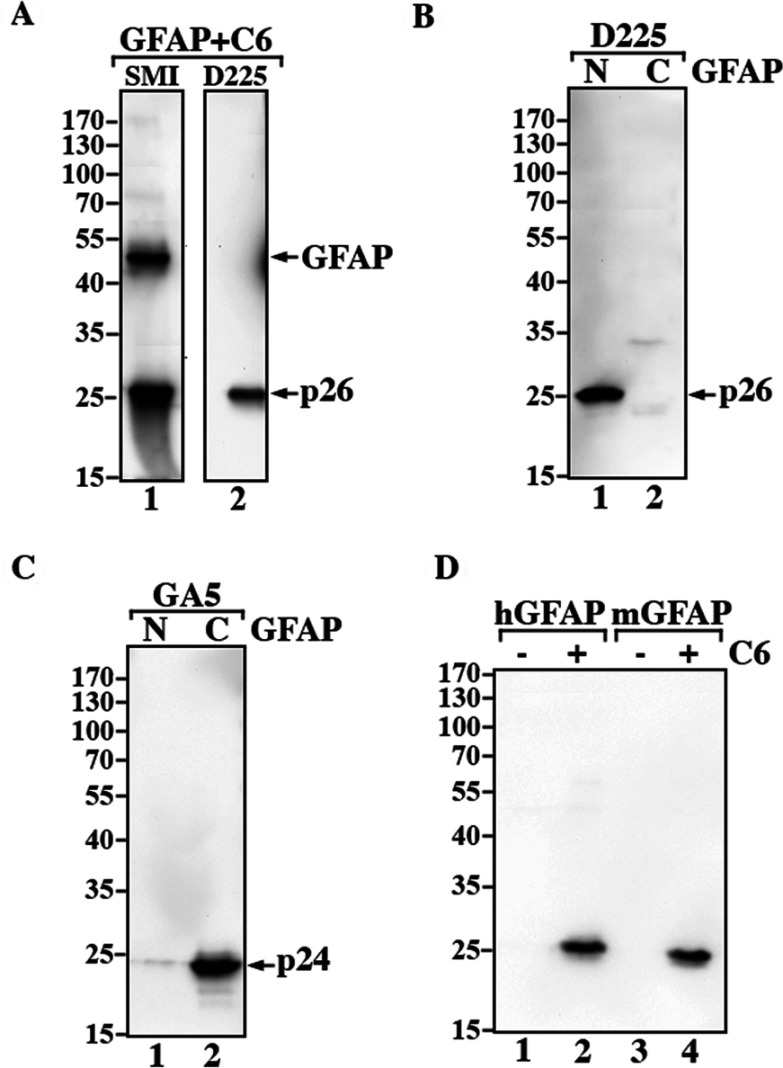
Characterization of caspase cleavage site-specific antibody to N-GFAP (**A**) Caspase-cleaved GFAP was analyzed by immunoblotting with the indicated antibodies. The SMI-21 antibody recognized both intact GFAP and N-GFAP (lane 1), whereas the D225 antibody recognized N-GFAP but not intact protein (lane 2). (**B**) The D225 antibody also recognized purified recombinant N-GFAP (lane 1) but not C-GFAP (**B**, lane 2), confirming the specificity of this antibody. (**C**) The presence of C-GFAP was demonstrated by immunoblotting with GA-5 antibody (lane 2). (**D**) Purified recombinant human (lane 1) and mouse GFAP (lane 3) digested with active caspase 6 (lanes 2 and 4) were analyzed by immunoblotting. Notice that the D225 antibody recognized caspase-generated N-terminal fragments from both human (lane 2) and mouse GFAP (lane 4).

Using an immunogen peptide containing the exposed VELD^225^ sequence, we generated a neo-epitope antibody, D225 that specifically recognized N-GFAP but not C-GFAP or intact GFAP (Figure 3A, cf. lanes 1 and 2). The specificity of this antibody was further confirmed by immunoblotting purified recombinant proteins. The D225 antibody recognized N-GFAP but not C-GFAP (Figure 3B). It could also recognize the caspase-generated N-terminal fragment of mouse GFAP (Figure 3D, lane 4), and the slightly faster electrophoretic mobility compared with human GFAP (Figure 3D, lane 2) likely reflecting sequence differences observed for full-length proteins (DeArmond et al., 1986). These data indicate epitope-sharing between VEMD and VELD by the D255 polyclonal peptide specific antibodies and confirm the VEXD consensus motif as the major caspase 6 cleavage site (Talanian et al., 1997; Nicholson, 1999).

**Figure 4:**
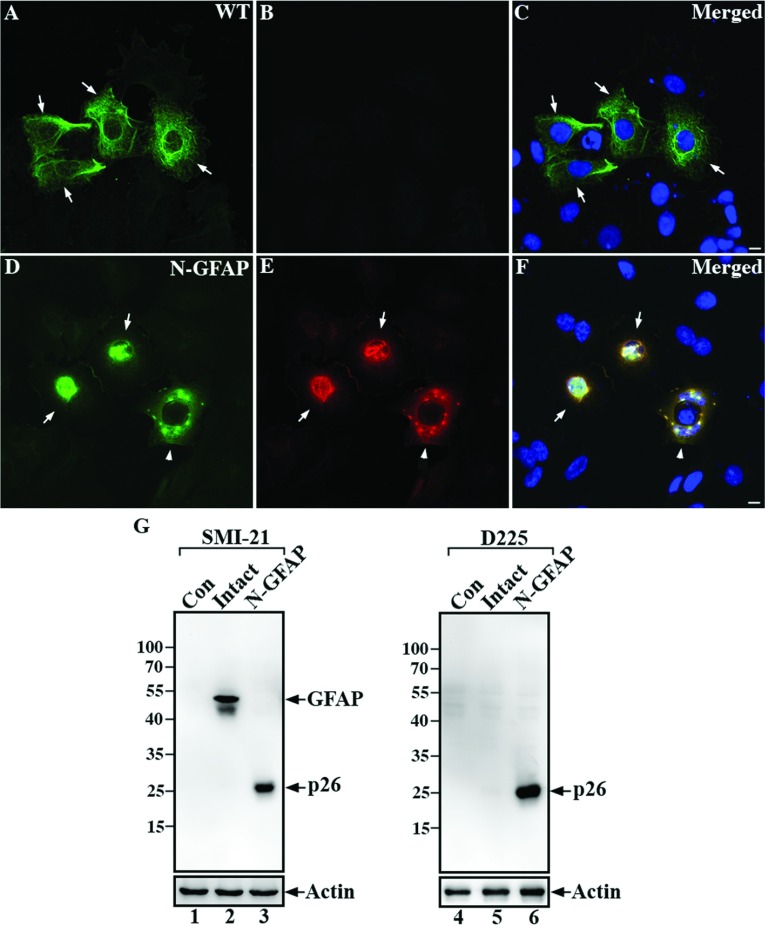
Detection of N-GFAP in MCF7 cells with the D225 antibody MCF7 cells were transiently transfected with either intact GFAP (**A**–**C**) or N-GFAP (**D**–**F**). At 48 h after transfection, the distribution of GFAP was visualized by double-label immunofluorescence microscopy using the SMI-21 (**A** and **D**, green channel) and D225 (**B** and **E**, red channel) antibodies. Merged images show the superimposition of the green and red signals, with overlapping areas appearing yellow (**C** and **F**). The nuclei were visualized by DNA staining with DAPI (**C** and **F**). When expressed in MCF7 cells, intact GFAP mainly formed extended filaments with some peripheral accumulations that were stained by the SMI-21 antibody (**A**, arrows) but not the D225 antibody (**B**). Transfecting N-GFAP into this cell line resulted in the formation of cytoplasmic aggregates that were stained by both the SMI-21 (**D**, arrows) and D225 antibodies (**E**, arrows). Occasionally, N-GFAP formed small clumps superimposed upon a diffused cytoplasmic staining pattern (**D** and **E**, arrowhead). Bar, 10 μm. (**G**) Total cell lysates prepared from untransfected cells (lanes 1 and 4) and cells transfected with either intact GFAP (lanes 2 and 5) or N-GFAP (lanes 3 and 6) were analyzed by immunoblotting with the SMI-21 (lanes 1–3) and D225 (lanes 4–6) antibodies. Immunoblots probed with anti-actin antibody were used as a loading control.

The D225 antibody was capable of recognizing N-GFAP in MCF-7 cells as well as in the astrocyte cell line U343MG that has well-established GFAP networks (Supplementary Figure S3 available at http://www.asnneuro.org/an/005/an005e125add.htm). Intact GFAP formed filaments and peripheral bundles that were readily stained in transiently transfected MCF7 cells by the commercial antibody SMI-21 (Figure 4A, arrows) but not by the D225 antibody (Figure 4B). When N-GFAP was transiently transfected alone, it formed aggregates that were easily stained by the D225 antibody (Figure 4E, arrows). Blotting data confirmed these immunofluorescence results (Figure 4G).

**Figure 5:**
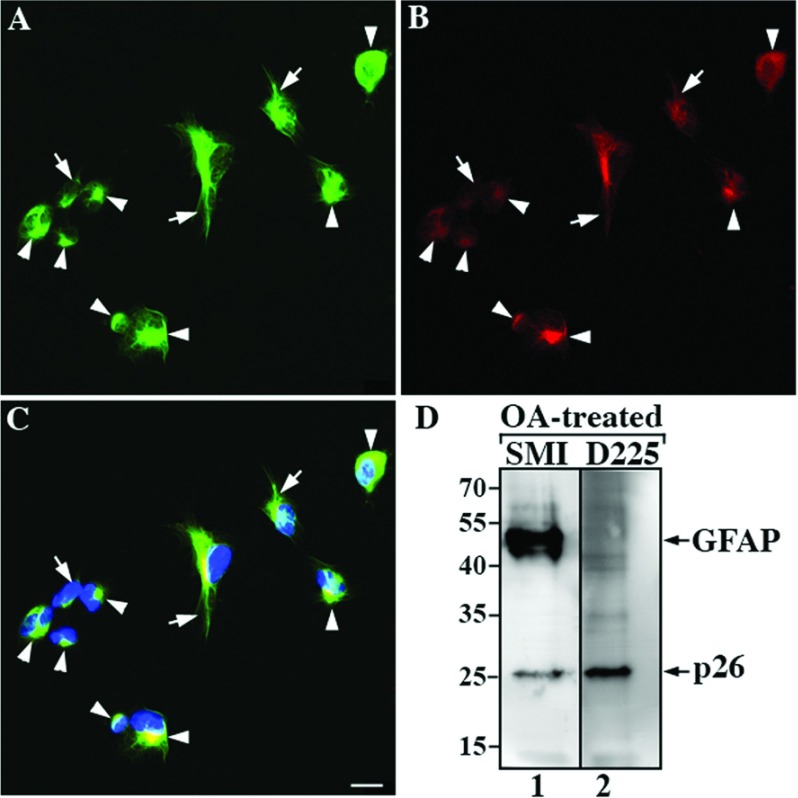
Caspase-mediated GFAP proteolysis in human astrocytoma U343MG cells treated with an apoptogen U343MG cells were treated with 200 nM OA for 4 h. OA-treated cells were fixed and processed for double label immunofluorescence microscopy using the anti-GFAP SMI-21 (**A**, green channel) and the fragment-specific D225 (**B**, red channel) antibodies. Merged image shows the superimposition of green and red signals with nuclear staining by DAPI (**C**). Notice that cells treated with OA resulted in the formation of bundled filaments (**A**–**C**, arrows) and cytoplasmic aggregates (**A**–**C**, arrowheads) that were immunostained with both SMI21 (**A**) and D225 (**B**) antibodies. Scale bars, 10 μm. (**D**) Analysis of total lysates prepared from OA-treated cells by immunoblotting with SMI-21 (lane 1) and D225 (lane 2) antibodies showed that cells treated with OA produced N-GFAP that was recognized by both antibodies.

U343MG cells treated for 4 h with 200 nM OA exhibited many morphological features of apoptosis including cellular rounding, cytosolic shrinkage as well as nuclear condensation and fragmentation (Figure 5C). Associated with these morphological changes were activation of caspase 3 and the cleavage of a well-characterized caspase 3 substrate PARP (poly(ADP-ribose) polymerase) into its characteristically sized cleavage products (Supplementary Figure S4 available at http://www.asnneuro.org/an/005/an005e125add.htm). This treatment also caused the collapse of the GFAP networks into filament bundles (Figure 5A–C, arrows) and perinuclear aggregates (Figure 5A–C, arrowheads). These structures were immunopositive for both N-GFAP (Figure 5B) and total human GFAP (Figure 5A). Immunoblotting of total cell lysates prepared from OA-treated cells showed that the D225 antibody specifically detected a degradation product of about 26 kDa (Figure 5D, lane 2), suggesting caspase-mediated breakdown of GFAP in cells after exposure to apoptotic stimuli. These data confirm the specificity of the D225 antibody and illustrate its usefulness to detect specifically the caspase-mediated cleavage of GFAP at both the biochemical and cellular levels.

### Caspase sensitivity of AxD causing GFAP mutants

**Figure 6:**
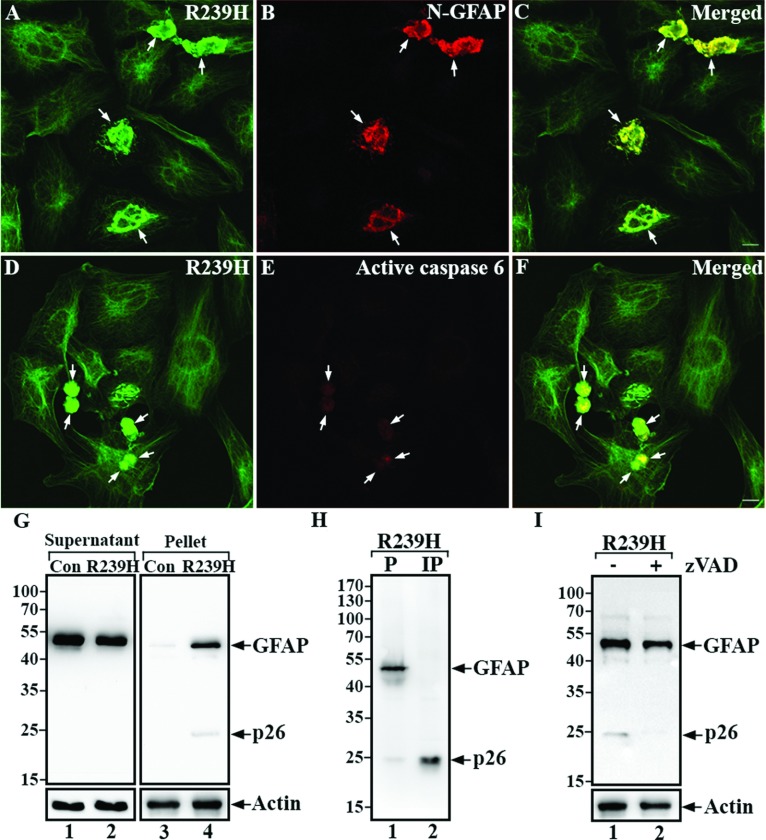
Caspase sensitivity of AxD-causing GFAP mutant U343MG cells transiently transfected with R239H GFAP were fixed at 48 h after transfection. Cells were processed for double-label immunofluorescence microscopy using SMI-21 anti-GFAP (**A** and **D**, green channel) and either D225 (**B**, red channel) or anti-active caspase 6 (**E**, red channel) antibodies. Merged image shows the region of colocalization appearing yellow (**C** and **F**). Notice that GFAP-rich aggregates in cells transfected with R239H GFAP (**A** and **D**, arrows) were also immunopositive for both N-GFAP (**B**, arrows) and active caspase 6 (**E**, arrows). Bar, 10 μm. (**G**) Supernatant (S) and pellet (P) fractions prepared from untransfected (lanes 1 and 3) or R239H GFAP-transfected (lanes 2 and 4) cells were analyzed by immunoblotting with the SMI-21 antibody. Notice a ~26 kDa degradation product was detected in the pellet fraction of R239H-transfected cells (lane 4). (H) The identity of this fragment was confirmed by immunoprecipitating N-GFAP from the pellet fraction (lane 1) with the D225 antibody, followed by immunoblotting with the SMI-21 antibody (lane 2). (I) Immunoblotting analysis of total cell lysates with the SMI-21 antibody showed that cells transfected with R239H GFAP generated N-GFAP (lane 1, p26), whose production was inhibited by the caspase inhibitor zVAD-fmk (lane 2).

U343MG cells were transiently transfected with R239H GFAP, one of the most common mutations associated with AxD. This induced GFAP-containing aggregates that were readily stained by the D225 antibody, suggesting that N-GFAP might be one of the components of GFAP-rich aggregates (Figures 6A–6C). We also examined the activation status of caspase 6 and found active caspase 6 localized at sites of GFAP aggregation (Figure 6, D–F). Immunoblotting of cell extracts from R239H-transfected cells detected a cleavage product similar in size to N-GFAP in the pellet fraction (Figure 6G, lane 4). The D225 antibody was able to immunoprecipitate N-GFAP from a solubilized pellet fraction (Figure 6H). Addition of a broad-spectrum caspase inhibitor, zVAD-fmk to cells transiently transfected with R239H GFAP reduced the abundance of the N-GFAP fragment (Figure 6I). These data suggest that R239H GFAP is susceptible to caspase cleavage in transiently transfected U343MG cells.

### Analysis of GFAP expression and degradation in mouse models of AxD

**Figure 7:**
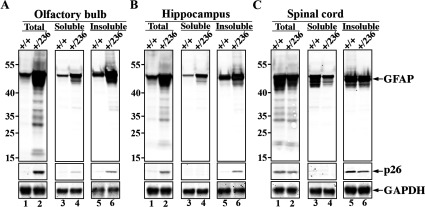
Analyses of GFAP expression and degradation in R236H/+ mice by immunoblotting Total (20 μg per lane), soluble (20 μg per lane) and insoluble (1.6 μg per lane) fractions were prepared from olfactory bulb (**A**), hippocampus (**B**) and spinal cord (**C**) of wild-type (+/+) and GFAP^+/R236H^ (+/236) mice. Samples were analyzed by immunoblotting using anti-GFAP GA-5 and D225 antibodies. Immunoblots were probed with an anti-GAPDH antibody to demonstrate equivalent protein loading of each lane. The relative electrophoretic mobility of molecular mass markers (in kDa) is indicated adjacent to lane 1. Experiments were performed with three pairs of wild-type and R236H/+ mice at 8 weeks of age.

Total protein lysates were prepared from different regions of the CNS, including olfactory bulb, hippocampus and spinal cord from both wild-type (GFAP^+/+^) and GFAP^+/R236H^ mice (Hagemann et al., 2006) with mutation corresponding to the R239H mutation found in human AxD. Immunoblotting analyses using GA-5 and D225 antibodies showed that the N-GFAP was detected only in olfactory bulb (Figure 7A, lane 2) and hippocampus (Figure 7B, lane 2) of GFAP^+/R236H^ mice but not wild-type controls (Figure 7A and B, lane 1,). In the spinal cord, however, the N-GFAP was detected in both wild-type and GFAP^+/R236H^ mice (Figure 7C, lanes 1and 2). When the tissue samples were fractionated into pellet and supernatant fractions, then N-GFAP was detected exclusively in the insoluble fractions of both the wild-type (Figure 7C, lane 5) and the GFAP^+/R236H^ (Figure 7A–C, lane 6) mice by the D225 antibody. These data confirm that the expression of GFAP varies considerably between different regions of the CNS (Jany et al., 2013). Importantly, they also suggest that the expression of R236H GFAP increases the caspase-mediated cleavage of GFAP.

**Figure 8:**
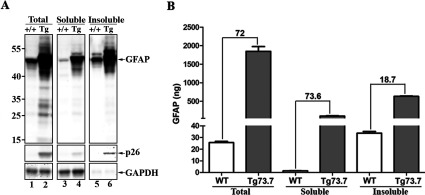
Elevated expression of human wild-type GFAP generates a caspase cleavage product in GFAP^Tg^ mice (**A**) Total (20 μg per lane), soluble (20 μg per lane) and insoluble fractions (1.6 μg per lane) prepared from whole brains of wild-type and GFAP^Tg^ mice were separated by SDS/PAGE followed by immunoblotting with antibodies to total GFAP, N-GFAP and GAPDH, which was used as a loading control. A duplicate gel stained with Coomassie Blue was also shown to assist comparison of equal protein loading (Supplementary Figure S5 available at http://www.asnneuro.org/an/005/an005e125add.htm). Molecular mass markers (in kDa) are labeled adjacent to lane 1. (**B**) GFAP protein levels were measured by quantitative ELISA in samples prepared from wild-type and GFAP^Tg^ mice (Tg73.7). GFAP^Tg^ mice showed a 72-fold increase in total GFAP, a 73.6-fold increase in soluble GFAP and a 18.7-fold increase in insoluble GFAP compared with wild-type controls. N **=** 4 for each group. Error bars indicate SD.

Similar analyses of transgenic mice (GFAP^Tg^) that constitutively overexpress human wild-type GFAP (Messing et al., 1998) revealed that caspase-mediated cleavage of GFAP was a feature of this model too. The presence of the caspase-produced N-GFAP was confirmed by immunoblotting analyses (Figure 8A). GFAP levels in GFAP^Tg^ mice were increased markedly with both the full length and GFAP degradation products being detected (Figure 8A, lane 2). Using GA-5 antibody, this marked increase in GFAP in both soluble and insoluble fractions of GFAP^Tg^ mice (Figure 8A, lanes 4 and 6) compared with wild-type controls (Figure 8A, lanes 3 and 5) was confirmed. The D225 antibody detected N-GFAP in samples from GFAP^Tg^ mice (Figure 8A, lane 2), but not from wild-type controls (Figure 8A, lane 1). As with the cell-based studies, N-GFAP was detected mainly in the insoluble fraction (Figure 8A, lane 6) consistent with its segregation into cytoplasmic aggregates. Quantitative ELISA (Figure 8B) revealed that GFAP protein levels were increased 72-fold in total brain lysates, 73.6-fold in the soluble fraction and 18.7-fold in the insoluble fraction of GFAP^Tg^ mice compared with wild-type controls. Therefore caspase-mediated GFAP proteolysis correlates with elevated GFAP in this mouse model of AxD.

### Potential functional consequence of caspase cleavage of GFAP

**Figure 9:**
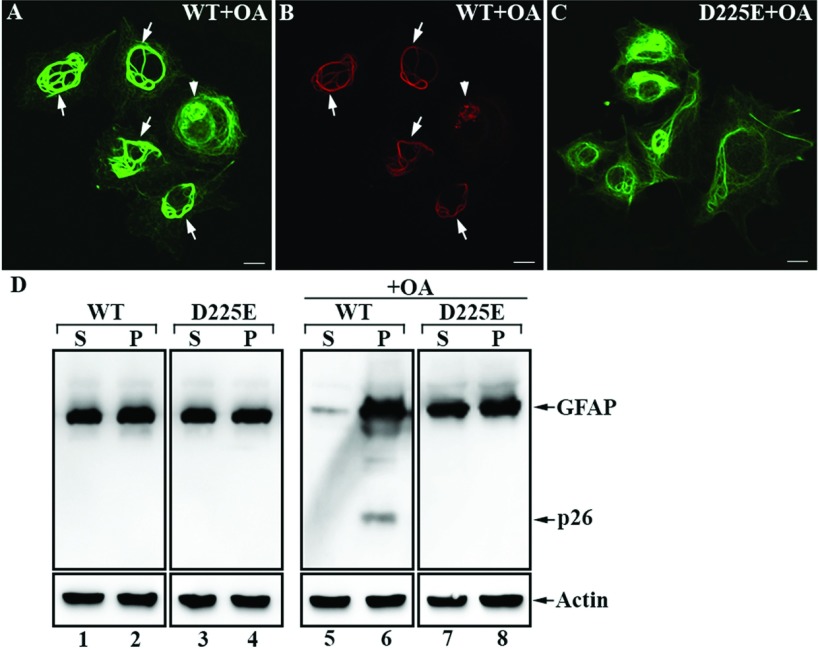
Effect of caspase cleavage on the solubility properties of GFAP MCF7 cells transiently transfected with either wild-type or D225E GFAP were treated with 200 nM OA for 2 h. After OA treatment, cells were fixed and processed for double label immunofluorescence microscopy using the anti-GFAP SMI-21 (**A** and **C**, green channel) and N-terminal fragment-specific D225 (**B**, red channel) antibodies. Bar, 10 μm. (**D**) Cells transfected with either wild-type or D225E GFAP were either untreated (lanes 1–4) or treated with OA (lanes 5–8). Cells were then extracted using harsh extraction buffer as described in the Materials and Methods, and the supernatant (S) and pellet (P) fractions were analyzed by immunoblotting with the SMI-21antibody. Blots probed with anti-actin antibody were used as a loading control. Notice that after OA-treatment wild-type GFAP, alone with its degradation products, was more resistant to extraction (lane 6) compared with the D225E mutant (lane 8).

To examine the functional consequence of caspase-mediated GFAP proteolysis at VELD^225^, MCF7 cells were transiently transfected with either wild-type GFAP or caspase cleavage-resistant D225E mutant. MCF7 cells were selected because they lack endogenous GFAP and caspase 3 (Janicke et al., 1998), thereby avoiding the potentially confounding influence of the endogenous caspase cleavage-sensitive GFAP at additional sites. When transfected into this cell line, wild-type and D225E GFAP formed filament networks in most of the transfected cells (results not shown). OA treatment caused the collapse of both wild-type (Figure 9A) and D225E (Figure 9C) GFAP filaments, but generated N-GFAP only in wild-type GFAP-transfected cells (Figure 9B). Double-label immunofluorescence microscopy demonstrated colocalization of the N-GFAP (Figure 9B) with the OA-collapsed GFAP aggregates (Figure 9A, arrowhead) and filament bundles (Figure 9A, arrows), indicating that the presence of N-GFAP could potentially influence GFAP filament properties. To test this hypothesis, we assessed biochemically the solubility properties of wild-type and D225E GFAP in transfected MCF7 cells. Using an extraction buffer containing deoxycholate (Perng et al., 2006), a significant proportion (~50%) of both wild-type and D225E GFAP could be extracted from transfected cells (Figure 9D, lanes 1 and 3). After OA treatment, the solubility of D225E GFAP was not dramatically altered (Figure 9D, lanes 7 and 8) compared with untreated cells (Figure 9D, lanes 3 and 4), whereas wild-type GFAP, alone with its degradation products, was more resistant to extraction (Figure 9D, lane 6). Taken together, these data suggest that caspase cleavage of GFAP is a functionally relevant proteolytic event that can potentially alter the solubility of GFAP filament.

## DISCUSSION

Caspases play an essential role during apoptotic cell death. These enzymes define a class of cysteine-aspartic acid proteases that posttranslationally modify their substrates through cleavage at a specific aspartic acid residue. Although caspase cleavage of several IF proteins, including the nuclear lamins, keratins, vimentin and desmin (Marceau et al., 2007), has been well documented, relatively few studies on caspase-mediated proteolysis of GFAP have been reported. Evidence for the proteolytic cleavage of GFAP by caspase 6 was provided by a recent study in human primary neurons (Klaiman et al., 2008), where a simple proteomic approach identified three potential caspase 6 cleavage sites in GFAP: VERD^142^, QEAD^177^ and VELD^225^. However, the direct cleavage of GFAP at these sites has not been confirmed. In this study, we performed an in-depth characterization of caspase-mediated cleavage of GFAP via the use of a caspase cleavage site-specific antibody, which we characterized and validated. We show here that GFAP is cleaved selectively by caspase 6 at a conserved Asp^225^ residue in its L12 linker domain *in vitro*. Caspase cleavage of GFAP at this site generated an N-terminal fragment that dramatically alters filament assembly both *in vitro* and in transfected cells, and does so in a dominant manner. The truncated protein can also disturb endogenous network of intact GFAP. In addition, we demonstrate the presence of N-GFAP in transfected cells expressing AxD-causing mutants and in two types of AxD models carrying either mutant GFAP or overexpressing human wild-type GFAP, suggesting that GFAP mutation and elevated expression correlate with caspase-mediated GFAP proteolysis. Finally, we provide evidence to show that caspase cleavage of GFAP has a functional impact on GFAP filament properties.

Our data differ from those reported by Mouser et al., 2006, who used a site-directed antibody to caspase-cleaved GFAP to demonstrate that GFAP is cleaved at DLTD^266^, a unique caspase cleavage site that is found only in GFAP and not in other IF proteins. A caspase cleavage product-specific antibody strongly stained degenerating astrocytes in AD brain and colocalized with an antibody specific to active caspase 3, providing evidence that the caspase-mediated cleavage of GFAP; activation of apoptotic pathways, and degeneration of astrocytes might be linked. In our *in vitro* cleavage assay, however, GFAP was resistant to caspase 3 cleavage (Supplementary Figure S6 available at http://www.asnneuro.org/an/005/an005e125add.htm) despite efficient cleavage by caspase 6. The inefficient cleavage of GFAP by caspase 3 may be explained by the fact that DLTD^266^ is part of a heptad repeat in the α-helical 2B subdomain, which is buried within the coiled-coil dimer making it inaccessible for cleavage. Another possible explanation is that our use of active caspase 3 utilized a concentration that was insufficient for *in vitro* activity. This is supported by the previous findings that caspase 6 can cleave its substrates at low doses, whereas caspase 3 is able to generate the characteristic cleavage patterns only at higher doses (Caulin et al., 1997; Schutte et al., 2004).

GFAP breakdown products could result from post mortem or *in vitro* degradation of the protein (DeArmond et al., 1983). GFAP is susceptible to cleavage by calcium-dependent proteases such as calpain (Lee et al., 2000), producing a range of breakdown products between 38 and 50 kDa (Zoltewicz et al., 2012). However, our data are not compatible with the possibility that the GFAP degradation products resulted from calpain-mediated digestion, as we took deliberate steps to avoid *in vitro* degradation by the activation of proteases during the sample preparation process. Indeed, we found that a 26 kDa proteolytic fragment was invariably produced when mutant forms of GFAP were transiently overexpressed in U343MG cells (Chen et al., 2011), but it was not detected in the untransfected cells that contained abundant endogenous GFAP. In addition to caspase and calpain, ubiquitin proteasome system is also involved in the proteolysis of GFAP, which regulates the turnover of the glial filaments (Tang et al., 2006; Tang et al., 2010). The signals, if any, that target GFAP for different degradation pathways and the significance of these proteolytic events have yet to be determined. Understanding the significance and regulation of GFAP proteolysis is important from a cell-biological standpoint and may also have pathophysiological relevance to degenerative disorders in which cytoskeletal defects result from GFAP proteolysis.

### Structural consequences of caspase-mediated GFAP proteolysis

Like other IF family members, GFAP has a characteristic tripartite domain structure consisting of a central α-helical rod domain flanked by non-helical head and tail domains. While the head domain of GFAP is essential for normal filament assembly (Quinlan et al., 1989), the tail domain plays an important role in controlling filament width *in vitro* (Quinlan et al., 1989; Ralton et al., 1994) as well as in establishing proper IF networks *in vivo* (Chen and Liem, 1994). Our *in vitro* studies demonstrate that C-GFAP lacking the head and coil 1 domains completely abolished its filament-forming ability, producing soluble complexes that remained in a discrete oligomeric state under all conditions tested. In contrast, N-GFAP missing the coil 2 and tail domains formed structures that were irregular in diameter and aggregation prone. These data suggest that while the N-terminal half of GFAP is essential for filament elongation, the C-terminal half is involved in controlling filament width and inter-filament interactions. Previous studies on type III IF proteins proposed that the C-terminal tail domain associates intramolecularly with the C-terminal end of the rod domain (Kouklis et al., 1991), which form a surface-exposed loop structure that prevents inappropriate subunit interactions in the assembly process and regulates filament thickness. This offers an explanation of the altered width of filaments assembled from truncated forms of vimentin (Herrmann et al., 1996; Herrmann et al., 2000) and the changed *in vitro* assembly characteristics of N-GFAP we report here.

This study parallels recent studies on a similarly truncated desmin, which contains the first 264 amino acids of desmin (N-desmin). Both N-GFAP and N-desmin have a strong tendency to aggregate *in vitro* (Bar et al., 2009) and in transfected cells (Chen et al., 2003), and both appear to function as potent disruptors of IF assembly. These findings are consistent with the observations that a similar N-terminal vimentin fragment comprising the head and conserved 1A domains interferes with normal filament assembly in a dominant-negative fashion (Kural et al., 2007; Chang et al., 2009).

### Functional consequences of caspase-mediated GFAP proteolysis

IFs fulfill dual roles in apoptosis by modulating events upstream of caspase activation, providing resistance to apoptosis initiation and by enhancing downstream caspase-mediated proteolysis to facilitate the execution of cell death (Marceau et al., 2007). Although several IF proteins, including keratin (Yoneda et al., 2004) and nestin (Sahlgren et al., 2006), exhibit anti-apoptotic properties, caspase cleavage of IF proteins could also generate pro-apoptotic fragments that amplify cell death signals (Byun et al., 2001). Our data show that caspase cleavage of GFAP generated two major proteolytic fragments, but neither of these fragments significantly induced apoptotic cell death when transiently overexpressed in either MCF7 or U343MG cells. These findings are in agreement with those of Chen et al., 2003, who demonstrated that cultured myoblasts transiently transfected with a similarly truncated desmin encoding amino acids 1**–**263 were still viable at 72 h, despite the presence of extensive aggregates. Although we cannot exclude the possibility that the presence of caspase cleavage products sensitizes cells to the induction of apoptosis by oxidative stress (Cho and Messing, 2009) or other apoptotic stimuli (Tang et al., 2006), our results argue against a direct pro-apoptotic function for either GFAP cleavage product. The general question that then confronts us is how caspase cleavage could influence the functional properties of GFAP filaments.

Our cellular models show that wild-type GFAP is more resistant to extraction compared with non-cleavable D225E mutant in OA-stressed MCF7 cells. The resistance of wild-type GFAP to extraction is likely due to the altered filament properties induced by the presence of caspase-cleaved GFAP fragment. *In vitro* studies provide additional evidence to support the role of caspase-produced GFAP fragment in promoting aggregate formation. These combined data suggest that caspase cleavage of GFAP has important functional consequences, decreasing GFAP solubility by changing filament-filament interactions in a manner that encourages aggregation. There are several other ways to modulate GFAP filament functional properties in astrocytes. They include phosphorylation (Sihag et al., 2007), the association of IF-associated proteins such as αB-crystallin (Koyama and Goldman, 1999; Perng et al., 1999), and the incorporation of other IF proteins, such as vimentin and nestin (Herrmann and Aebi, 2000). The proteolytic GFAP fragment produced on caspase cleavage provides a complementary mechanism that could potentially alter GFAP filament organization and filament properties in astrocytes.

## Online data

Supplementary data
