# Supplementary material for: Caspase cleavage of GFAP produces an assembly-compromised proteolytic fragment that promotes filament aggregation
Source: ASN Neuro. 2013 Nov 19;5(5):e00125. doi: 10.1042/AN20130032 (PMC3833455; doi:10.1042/AN20130032)
Supplement: Supplementary data [file an005e125add.pdf]

ASN NEURO RESEARCH ARTICLE

ASN NEURO 5(5):art:e00125.doi:10.1042/AN20130032

OPEN ACCESS

Caspase cleavage of GFAP produces an assembly-compromised proteolytic fragment that promotes filament aggregation

Mei-Hsuan Chen*, Tracy L. Hagemann†, Roy A. Quinlan‡, Albee Messing†§ and Ming-Der Perng*[1]
*Institute of Molecular Medicine, College of Life Sciences, National Tsing Hua University, Hsinchu, Taiwan.
†Waisman Center, University of Wisconsin, WI, USA
‡School of Biological and Biomedical Sciences, The University of Durham, UK
§Department of Comparative Biosciences, University of Wisconsin, WI, USA

SUPPLEMENTARY DATA

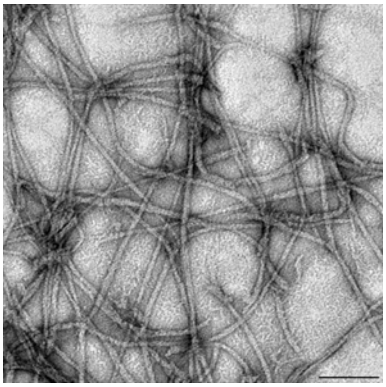

Figure S1 Effect of N-GFAP on the *in vitro* assembly of intact GFAP
Purified intact GFAP was coassembled with N-GFAP in 90:10 ratio. After assembly, samples were negatively stained and visualized by electron microscopy. Notice that inclusion of 10 % N-GFAP in the assembly mixture did not dramatically alter the morphology of the assembled filaments. Bar, 200 nm

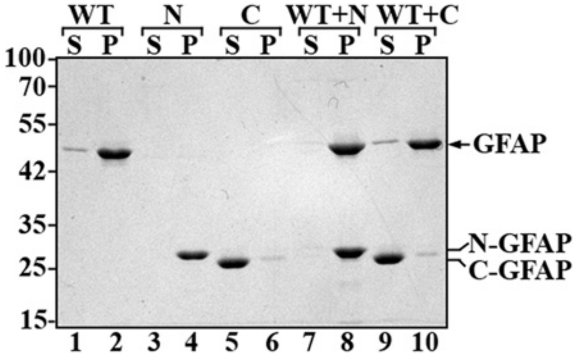

Figure S2 High-speed sedimentation assay confirmed that GFAP assembled efficiently
Intact, N- and C-GFAP were assembled either alone or in combinations with intact GFAP at a 75:25 ratio. Assembly products were subjected to high-speed sedimentation assay and the supernatant (S) and pellet (P) fractions were analyzed by SDS-/PAGE, followed by Coomassie Blue staining. A representative gel is shown, and the positions of N-, C- and intact GFAP are indicated. Molecular mass markers (in kDa) are labeled adjacent to lane 1. Under these assay conditions, most (>90 %) of the assembled intact GFAP was found in the pellet fraction (lane 2), confirming that intact GFAP had assembled efficiently. N-GFAP was also detected in the pellet fraction (lane 4). When coassembed with intact GFAP, N-GFAP was found exclusively in the pellet fraction (lane 8). In contrast, C-GFAP, either on its own (lane 5) or in combination with intact GFAP (lane 9), remained soluble and its presence did not affect the sedimentation behavior of intact GFAP (lane 10).

[1]To whom correspondence should be addressed (email mdperng@life.nthu.edu.tw).
© 2013 The Author(s) This is an Open Access article distributed under the terms of the Creative Commons Attribution Licence (CC-BY) (http://creativecommons.org/licenses/by/3.0/) which permits unrestricted use, distribution and reproduction in any medium, provided the original work is properly cited.

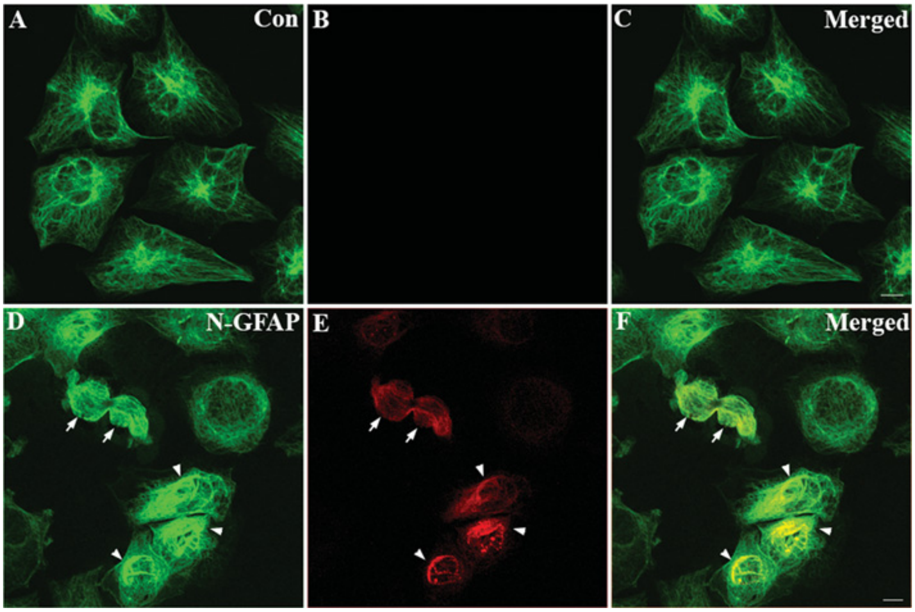

Figure S3 **Transient expression of N-GFAP resulted in the formation of cytoplasmic aggregates in human astrocytoma U343MG cells**
U343MG cells were either untransfected (**A**--**C**) or transfected with N-GFAP (**D** – **F**). At 48 h after transfection, cells were fixed and processed for double-label immunofluorescence microscopy using SMI-21 (**A** and **D**, green channel) and D225 (**B** and **E**, red channel) antibodies. Merged image shows the region of colocalization appearing yellow (**C** and **F**). Notice that while SMI-21 antibody readily stained endogenous GFAP in untransfected cells (**A**), no signal was produced by the D225 antibody (**B**). The D225 antibody strongly stained cells that were transfected with N-GFAP (**E**). The staining was apparent both along filament bundles (**E**, arrowheads) and in GFAP-rich aggregates (**E**, arrows) and largely colocalized with staining of total GFAP (**D**). Bar, 10 μm.

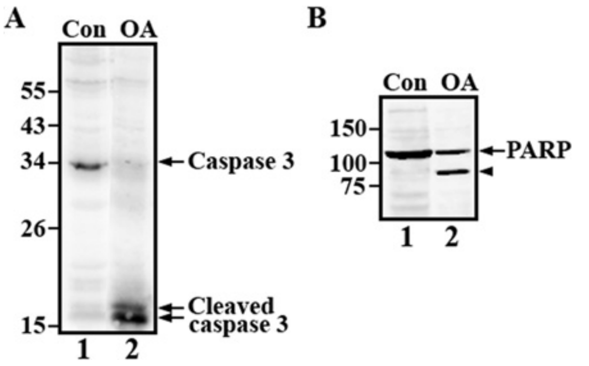

Figure S4 Activation of caspase 3 and cleavage of PARP in OA treated cells
U343MG cells were treated with 200 nM OA or equivalent volume of DMSO (Con) for 4 h. Total cell lysates prepared from control (**A** and **B**, lane 1) or OA-treated (**A** and **B**, lane 2) cells were analyzed by immunoblotting with antibodies to either caspase 3, including one to detect activated caspase 3 (**A**) or -PARP (**B**). Notice that cleaved caspase 3 (**A**, lane 2) and cleaved PARP fragment (**B**, lane 2, arrowhead) were detected in OA-treated cells.

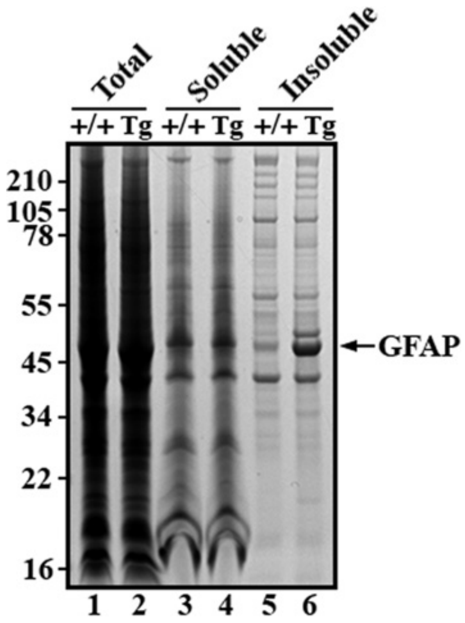

Figure S5 Analysis of protein expression in brains of GFAP^{Tg} mice
Total (20 μg per lane), soluble (20 μg per lane) and insoluble (1.6 μg per lane) fractions prepared from whole brains of wild-type (+/+, lanes 1, 3 and 5) and GFAP^{Tg} (Tg, lanes 2, 4 and 6) mice were analyzed by SDS-/PAGE and visualized by Coomassie Blue staining. The relative electrophoretic mobility of molecular mass markers (in kDa) is indicated adjacent to lane 1. The position of GFAP is indicated.

© 2013 The Author(s) This is an Open Access article distributed under the terms of the Creative Commons Attribution Licence (CC-BY) (http://creativecommons.org/licenses/by/3.0/) which permits unrestricted use, distribution and reproduction in any medium, provided the original work is properly cited.

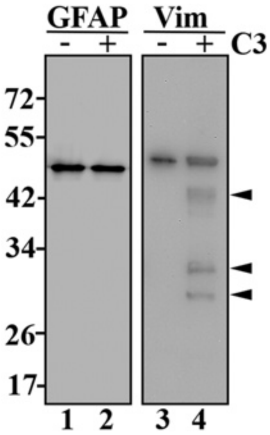

Figure S6 GFAP is not cleaved by caspase-3 *in vitro*
Purified recombinant GFAP (lanes 1 and 2) and vimentin (lanes 3 and 4) were incubated with buffer alone (lanes 1 and 3) or 2.5 U of purified active caspase-3 (C3, lanes 2 and 4) for 1 h at 37 °C. The reaction products were analyzed by immunoblotting using anti-GFAP (SMI-21) and anti-vimentin (V9) antibodies. Notice that vimentin was cleaved by caspase 3 into several proteolytic fragments *in vitro* (lane 4, arrowheads), whereas GFAP was resistant to caspase 3 cleavage (lane 2).

© 2013 The Author(s) This is an Open Access article distributed under the terms of the Creative Commons Attribution Licence (CC-BY) (http://creativecommons.org/licenses/by/3.0/) which permits unrestricted use, distribution and reproduction in any medium, provided the original work is properly cited.
